# Pregnancy Outcomes in Women Following a Pelvic Ring Injury: A Retrospective Cohort Study Involving 120 Births

**DOI:** 10.2106/JBJS.OA.26.00327

**Published:** 2026-11-11

**Authors:** Reagan Mead, Susan Lewis, Emily R. Oleisky, Juanita Velasco-Castro, Andres Fidel Moreno Diaz, Chelsea Fechter, Lauren Luther, Phillip M. Mitchell, Lauren M. Tatman

**Affiliations:** 1 Department of Orthopaedic Surgery, Vanderbilt University Medical Center, Nashville, Tennessee; 2 Department of Obstetrics and Gynecology, Vanderbilt University Medical Center, Nashville, Tennessee; 3 Vanderbilt School of Medicine, Nashville, Tennessee

## Abstract

**Background:**

Pelvic ring injuries can alter pelvic shape and mobility. Residual deformity or constraints from surgical implants may impair the natural progression of childbirth. Prior studies suggest an increased rate of cesarean section (CS) after pelvic trauma, but it remains unclear whether cephalopelvic disproportion drives this finding. The primary objective of this study was to determine the rate of CS attributed to cephalopelvic disproportion or arrest of labor in patients with a history of pelvic ring injury. The secondary objective was to determine the difference in rates of CS in these patients based on the presence of joint-spanning surgical implants and residual post-traumatic deformity.

**Methods:**

A retrospective cohort study was performed at a Level I trauma center that provides comprehensive obstetric care. Female patients with prior pelvic ring injury (AO/OTA 61B, 61C) and subsequent live childbirths between 2003 and 2024 were identified. Inclusion criteria were age ≥18 years at delivery, injury preceding pregnancy, and adequate imaging and obstetric records. Post-traumatic pelvic ring deformity and shape were evaluated based on radiographic measurements incorporating obstetrical and orthopaedic trauma concepts. A certified midwife classified CS indications based on national guidelines.

**Results:**

Among 87,159 deliveries, 120 live births to 81 eligible patients were identified. The CS rate was 59% in patients with prior pelvic ring injuries versus 36% overall (p < 0.001). Of the 120 deliveries, 57 had a scheduled CS and 63 underwent a trial of labor. Of those who underwent a trial of labor, 49 (77.8%) had a vaginal delivery and 14 (22.2%) were transitioned to CS for fetal indications rather than the arrest of labor. Neither joint-spanning implants (aRR 1.20, 95% CI 0.90-1.61) nor pelvic ring post-traumatic deformities (aRR 1.00, 95% CI 0.75-1.35) were independently associated with CS.

**Conclusions:**

The elevated CS rate after pelvic ring injury was not associated with joint-spanning implants or measured residual pelvic deformity, and nearly one-third of patients lacked a clear CS indication. These findings highlight opportunities to reduce potentially unnecessary CS through multidisciplinary collaboration.

**Level of Evidence:**

Level III. See Instructions for Authors for a complete description of levels of evidence.

## Introduction

Pelvic ring injuries can result in long-term functional impairments among survivors^1^. These injuries may also negatively affect future pregnancy outcomes. Multiple studies have reported high rates of cesarean section (CS) in women with a prior pelvic ring injury, ranging from 48% to 62%^2-4^. This is considerably higher than the 32% rate for all women in the United States^5^. It is unknown if the increased rate of CS is related to altered pelvic anatomy associated with the prior pelvic ring injury, patient or physician preference, or obstetric conditions and comorbidities. No guidelines currently exist for counseling these patients on future pregnancy risks and options.

Although CS is a life-saving intervention when medically indicated, it is associated with higher mortality and morbidity compared with vaginal delivery (VD)^5,6^. National and global CS rates have increased substantially in recent decades, and overuse has emerged as a major maternal safety concern^5,6^. Short-term maternal risks include increased blood loss, infection, and venous thrombosis, while long-term risks in subsequent pregnancies include abnormal placentation, increased risk of hemorrhage, uterine rupture, and hysterectomy^5,6^. In addition, growing evidence suggests that infants born through CS may face increased health risks compared with infants born vaginally^6^.

The American College of Obstetricians and Gynecologists (ACOG) describes indications for CS within clinical contexts^7^. An arrest of labor is the indication most likely to be influenced by a prior pelvic ring injury, given the potential for associated cephalopelvic disproportion. Following a pelvic ring injury, residual deformity and decreased mobility of the pubic symphysis or sacroiliac (SI) joints due to joint-spanning implants could contribute to cephalopelvic disproportion, which occurs when the shape of the pelvis does not allow the fetus to pass through the birth canal.

The primary objective of this study was to determine the rate of CS secondary to the arrest of labor in patients with a history of pelvic ring injury. Secondary objectives included the following: compare post-traumatic CS rates with the overall CS rate for women delivering a child at a single institution, determine the difference in rates of CS in women with a history of pelvic ring injury with and without joint-spanning (pubic symphysis or SI joint) surgical implants, and determine the difference in rates of CS in women with a history of pelvic ring injury based on residual post-traumatic deformity. We hypothesized that the arrest of labor due to cephalopelvic disproportion will be an uncommon occurrence in women delivering a child following a pelvic ring injury.

## Methods

A retrospective cohort study was performed at a Level I trauma center that provides comprehensive obstetrical care for routine and high-risk pregnancies in the region. Following Institutional Review Board approval, female patients with a history of a pelvic ring injury (AO/OTA 61B, 61C) who had a subsequent pregnancy with delivery at our institution from 2003 to 2024 were identified. The electronic medical record (EMR) and imaging were reviewed to determine the eligibility. Inclusion criteria were age ≥18 years at the time of delivery, pregnancy resulting in a live birth, pelvic ring injury preceding pregnancy(ies), and pelvis radiographs available for review. Exclusion criteria included miscarriage or stillbirth, pregnancy(ies) before or present at the time of pelvic ring injury, and pelvic ring injuries with an intact posterior arch (AO/OTA 61A).

Demographic, injury, treatment, and obstetric data were collected from the EMR. Body mass index (BMI) was calculated using the patient’s weight and height at the time of pelvic ring injury to avoid the pregnancy-related variation. All other dynamic demographic data were obtained for each pregnancy.

Pelvic ring injury details were obtained by EMR and radiographic review. The pubic symphysis or SI joint(s) were defined as stabilized if crossed by rigid fixation (plate or screw) at the time of delivery, accounting for any implant removal surgeries.

Post-traumatic pelvic ring deformity and shape were evaluated using 4 radiographic measurements: obstetric conjugate, interspinous diameter, pelvic asymmetry value, and vertical displacement (Fig. 1). These 4 measurements were used to incorporate obstetric pelvimetry concepts for identifying women at risk of cephalopelvic disproportion (Fig. 2) and orthopaedic concepts that have previously described post-traumatic deformity of the pelvic ring. Post-traumatic deformity of the pelvic ring was defined as meeting any of the following criteria: obstetric conjugate <10 cm, interspinous diameter <9.5 cm, pelvic asymmetry value ≥ 1 cm, or vertical displacement ≥1 cm^8-11^. Radiographic measurements were performed by an orthopaedic surgeon blinded to the pregnancy outcomes.

**Fig. 1:**
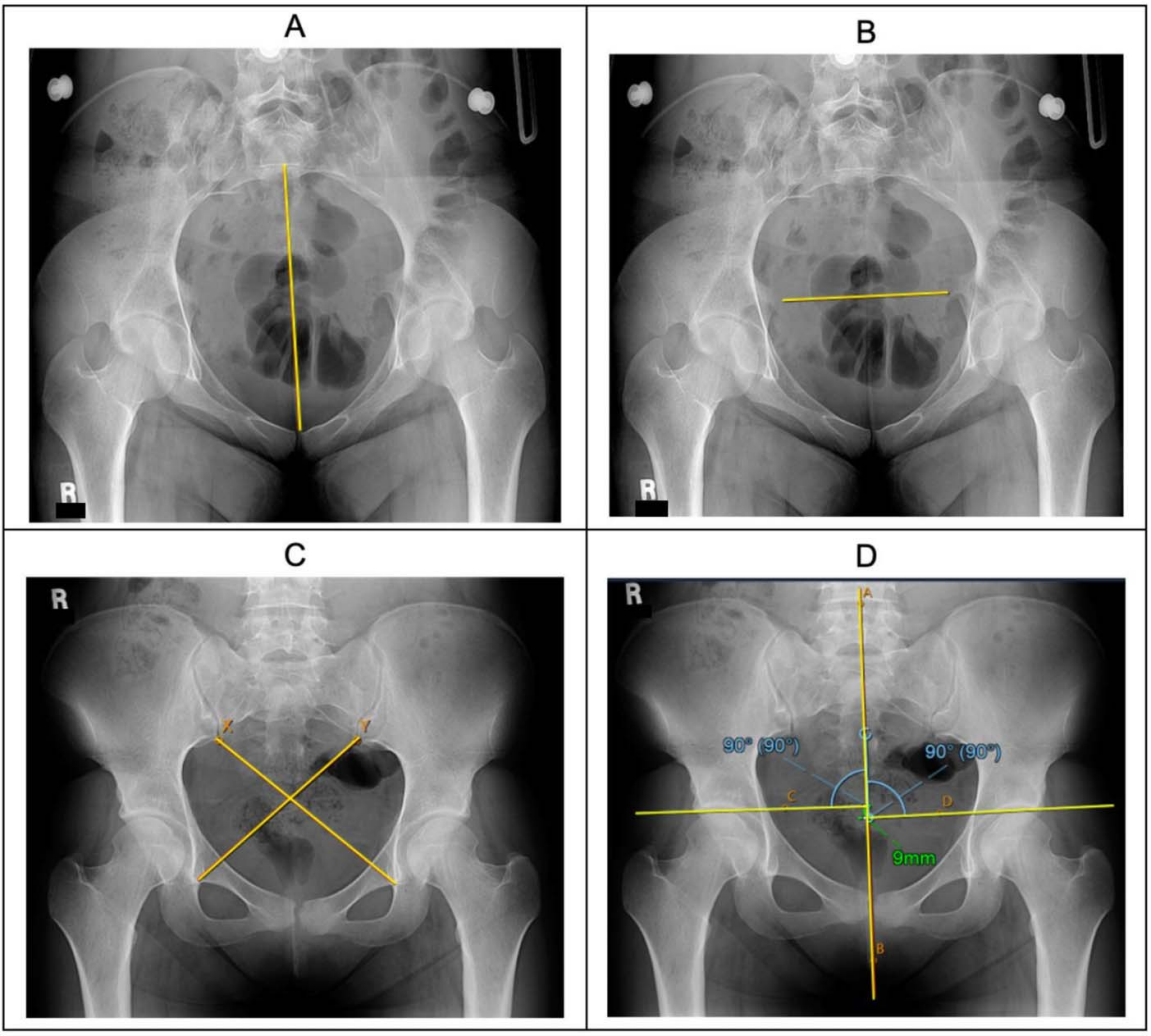
Radiographic measurements used to evaluate post-traumatic pelvic ring deformity and shape. **Fig. 1-A** Obstetric conjugate is measured on an inlet view of a pelvis radiograph and is the distance between the sacral promontory to the posterior aspect of the pubic symphysis. **Fig. 1-B** Interspinous diameter is measured on an inlet view of a pelvis radiograph and is the distance between the ischial spines. **Fig. 1-C** Pelvic asymmetry value is measured on an anteroposterior view of the pelvis, and it is the difference between the distance from the inferior aspect of the sacroiliac joint to the inferior aspect of the contralateral teardrop of one hemipelvis (X) and the contralateral hemipelvis (Y). **Fig. 1-D** Vertical displacement is measured on an anteroposterior view of the pelvis, and it is the distance between lines drawn from the top of each femoral head (Lines C-D) that are right angles from a vertical plumb line (Line A-B).

**Fig. 2:**
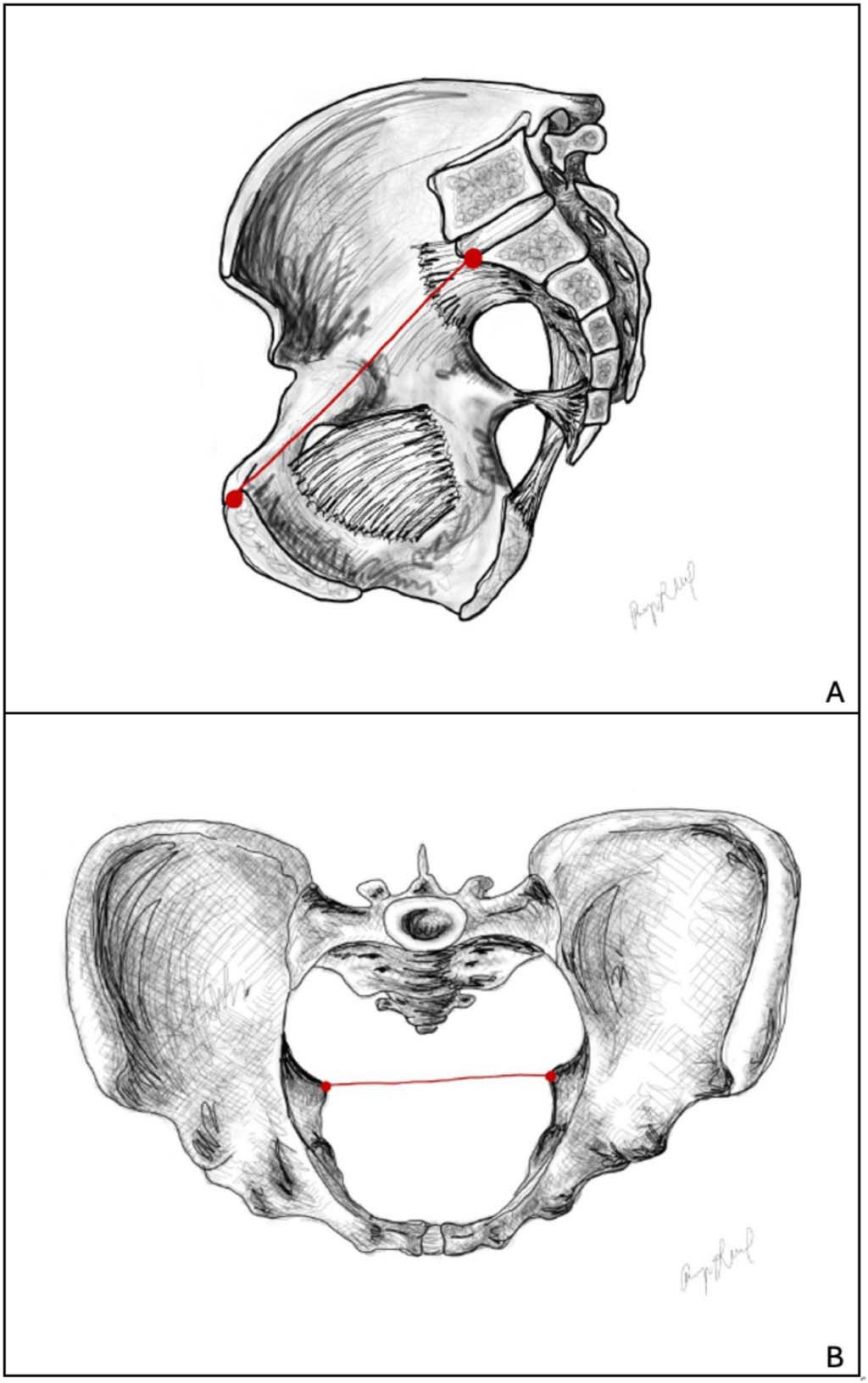
Obstetric pelvimetry measurements. Obstetric pelvimetry is the assessment of the maternal bony pelvis to estimate its size and shape, aiming to determine if it is adequate for vaginal delivery. **Fig. 2-A** Obstetric conjugate represents the smallest anteroposterior dimension of the bony pelvis. The critical limit value, which indicates a high likelihood for cephalopelvic disproportion, has been described as < 10 centimeters. **Fig. 2-B** Interspinous diameter represents the smallest transverse dimension of the bony pelvis with a critical limit value described as < 9.5 centimeters.

Pregnancy and delivery details were obtained by EMR review. A certified nurse midwife reviewed delivery records to determine indications for CS, which were categorized as absolute or relative based on national ACOG guidelines.

Continuous variables were summarized using medians with interquartile ranges. Bootstrap quantile regression was performed to obtain the effect estimates with 95% confidence intervals (95% CI). Effect estimates for continuous variables were obtained by bootstrap quantile regression with 2,000 replications and are reported as median differences with 95% CI. Categorical variables were presented as counts and percentages. For binary variables, the effect estimates are reported as relative risks (RR) with 95% CI. Multicategory variables and variables with sparse data were evaluated using the Fisher exact test.

CS rates among women with a history of pelvic ring injury were compared with those of the overall institutional obstetric population using chi-square testing. Unadjusted RR and corresponding 95% CI were calculated to quantify differences in CS rates.

Given the limited sample size, multivariable analyses were restricted to clinically relevant factors for CS, including maternal age and parity. Adjusted relative risks (aRR) and 95% CI were estimated using modified Poisson regression with robust variance estimation. Separate models were developed to assess the independent associations of joint-spanning implant and pelvic ring post-traumatic deformity with CS. In addition, a prespecified sensitivity analysis restricted to women without a history of CS was performed to evaluate factors associated with primary CS. The results were interpreted primarily using effect estimates and 95% CI. For RR, CI excluding 1.0 were considered evidence of association. For median differences, CI excluding 0 were considered evidence of a difference between groups. P values are presented as supplementary measures of statistical significance. All analyses were performed using STATA Version 18.0 (StataCorp LLC, College Station, TX).

## Results

From 2003 to 2024, 87,159 deliveries resulting in live births occurred at our institution with a CS rate of 36.1% (annual range 30.8-39.2%). The study cohort included 120 live births in 81 women that had sustained a prior pelvic ring injury and met inclusion criteria. Over the 22-year period, women with a history of a pelvic ring injury had a CS rate of 59.2% (Table 1), which was significantly higher than the overall CS rate during this time period (RR = 1.64; 95% CI 1.41-1.91; p <0.001).

**Table 1:** Delivery volume and Cesarean Section (CS) rate* per Year for All Women and for Women with a History of Pelvic Ring Injury Delivering at a Single Institution

Year	All Women		Women with Pelvic Ring Injury	
	Delivery Volume	CS Rate	Delivery Volume	CS Rate
2003	2,134	33.1%	1	100%
2004	2,514	32.3%	1	0
2005	2,885	32.7%	2	50%
2006	3,108	32.4%	4	0
2007	2,728	31.7%	2	50%
2008	2,682	30.8%	4	50%
2009	2,691	35.5%	5	60%
2010	3,497	36.8%	5	40%
2011	3,969	36.3%	5	20%
2012	4,389	36.0%	2	0
2013	4,513	35.3%	12	66.7%
2014	4,473	38.1%	6	83.3%
2015	4,233	36.5%	6	100%
2016	4,261	36.3%	5	40%
2017	4,778	37.3%	8	62.5%
2018	4,355	37.2%	9	44.4%
2019	4,414	35.6%	6	66.7%
2020	4,448	37.9%	5	80%
2021	5,128	37.7%	10	70%
2022	5,238	39.2%	11	63.6%
2023	5,203	37.7%	7	57.1%
2024	5,518	38.2%	4	100%
Total	87,159	36.1%	120	59.2%

* CS rate is for all women with delivery resulting in a live birth at our institution, regardless of gestational age and parity.

Among the 120 pregnancies in women with prior pelvic ring injury, 49 deliveries were VD and 71 were CS. Women who delivered via CS were older at the time of delivery, had a higher BMI, had higher rates of pregnancy-related complications, and, more frequently, had an ASA classification of 3 or greater compared with women who delivered via VD (Table 2).

**Table 2:** Patient, Injury, and Obstetric Characteristics*

	Vaginal Delivery	Cesarean Section	Effect Estimate (95% CI)
N	49	71	
Patient characteristics			
Age	25 (22-32)	29 (25-33)	Median difference 4.00 (1.00-7.00)
BMI	25.4 (23-28.3)	29.3 (25.7-37.2)	Median difference 3.80 (0.55-7.05)
Race			0.816
White	37 (75.5%)	57 (80.3%)	
Black	8 (16.3%)	9 (12.7%)	
Other	4 (8.2%)	5 (7.0%)	
ASA classification			RR 1.60 (1.23-2.08)
1-2	48 (98.0%)	62 (87.3%)	
≥ 3	1 (2.0%)	9 (12.7%)	
Smoking status			0.501
Current	13 (26.5%)	13 (18.3%)	
Former	6 (12.2%)	12 (16.9%)	
Never	30 (61.2%)	46 (64.8%)	
Current drug use			RR 0.77 (0.48-1.24)
No	38 (77.5%)	61 (85.9%)	
Yes	11 (22.4%)	10 (14.1%)	
Injury characteristics			
Injury severity score	17 (9-21)	17 (9-26)	Median difference 0.00 (-5.60-5.60)
Young-burgess classification			
LC 1	37 (75.5%)	52 (73.2%)	RR 0.95 (0.69-1.33)
LC 2	4 (8.2%)	11 (15.5%)	RR 1.28 (0.91-1.82)
LC 3	8 (16.3%)	3 (4.2%)	RR 0.44 (0.16-1.16)
Vertical shear	0	5 (7.0%)	0.078
Fracture type			0.513
Closed	49 (100%)	69 (97.2%)	
Open	0	2 (2.8%)	
Treatment of pelvic ring injury			RR 1.30 (0.97-1.74)
Nonoperative	34 (69.4%)	38 (53.5%)	
Operative	15 (30.6%)	33 (46.5%)	
Joints stabilized			0.592
0	34 (69.4%)	40 (56.3%)	
1	8 (16.3%)	15 (21.1)	
2	7 (14.3%)	16 (22.5)	
3	0	0	
Pelvic ring post-traumatic deformity			RR 0.99 (0.74-1.34)
No	24 (49.0%)	35 (49.3%)	
Yes	25 (51.0%)	36 (50.7%)	
Obstetric conjugate <10 cm	0	0	
Interspinous diameter < 9.5 cm	0	2 (2.8%)	
Pelvic asymmetry value ≥ 1 cm	21 (42.8%)	26 (36.6%)	
Vertical displacement ≥ 1 cm	13 (26.5%)	20 (28.2%)	
Obstetric characteristics			
Gestational age (weeks)	39.1 (38-40.1)	39.0 (37-39.1)	Median difference 0.14 (−1.10 to 0.81)
Birth weight (grams)	3,370 (2,945-3,584)	3,055 (2,450-3,401)	Median difference 315 (−623.04 to −6.96)
Parity			RR 1.18 (0.84-1.67)
Nulliparity	18 (36.7%)	20 (28.2%)	
Multiparity	31 (63.3%)	51 (71.8%)	
Prior cesarean section			<0.001
No	49 (100%)	41 (57.75%)	
Yes	0	30 (42.25%)	
Pregnancy complications			RR 1.44 (1.09-1.89)
No	42 (85.7%)	48 (67.6%)	
Yes	7 (14.2%)	23 (32.4%)	
Preterm labor	3 (6.1%)	4 (5.6%)	
Gestational diabetes	1 (2.0%)	6 (8.5%)	
Pre-eclampsia	2 (4.1%)	6 (8.5%)	
Gestational hypertension	1 (2.0%)	10 (14.1%)	
Other†	1 (2.04%)	4 (5.6%)	
Maternal postpartum complications			RR 1.13 (0.63-2.04)
No	47 (95.9%)	67 (94.4%)	
Yes‡	2 (4.1%)	4 (5.6%)	

* Values are given as the number of patients, with or without the percentage in parentheses, or as the median with interquartile range. Bootstrap quantile regression was performed to obtain the effect estimates with 95% confidence intervals (CI). Nonbinary categorical variables and variables with sparse data were evaluated using the Fisher exact test to obtain a p-value.

† Other pregnancy complications included deep venous thrombosis (1), fetal growth restriction (1), polyhydramnios (1), small bowel obstruction (1), and fetal hydrops (1).

‡ Maternal postpartum complications included ileus (1), postpartum hemorrhage (2), and surgical site infection/bacteremia/sepsis (3).

Of the 120 deliveries, 57 had a scheduled CS and 63 underwent a trial of labor. Of those who underwent a trial of labor, 49 (77.8%) had a VD and 14 (22.2%) were transitioned to CS for different indications. For the 71 CS deliveries, 20 (28.2%) had no identifiable indication and no CS deliveries were indicated due to the arrest of labor (Table 3). Of the 48 deliveries in women who had operative management of their pelvic ring, there were 15 VD, 30 scheduled CS, and 3 who had a trial of labor with conversion to CS (Appendix).

**Table 3:** Indications for Cesarean Section*

Absolute indication	10 (14.1%)
Fetal malpresentation (breech, transverse, or oblique lie)	4
History of classical cesarean	1
Umbilical cord prolapse	2
Monochorionic-monoamniotic twins	1
Placenta previa	1
History of myomectomy	1
Relative indication	41 (57.8%)
Non-reassuring fetal heart rate tracing	12
Previous cesarean section	29
Labor arrest	0
None†	20 (28.2%)

* Values are given as the number of patients, with or without the percentage in parentheses.

† Did not meet any criteria for CS indications described by ACOG.

After adjusting for age and parity, neither joint-spanning implants (aRR 1.20, 95% CI 0.90-1.61) nor pelvic ring post-traumatic deformities (aRR 1.00, 95% CI 0.75-1.35) were independently associated with CS. Among women without a prior CS, neither joint-spanning implants (aRR 1.16, 95% CI 0.74-1.82) nor pelvic ring post-traumatic deformities (aRR 1.06, 95% CI 0.68-1.64) were independently associated with primary CS delivery (Supplementary Table 1).

## Discussion

In the largest study to date evaluating pregnancy outcomes in women with a prior pelvic ring injury, the rate of CS was significantly higher than the general population. Despite this difference, our findings suggest that the increased CS rate is not driven by anatomic sequelae of the pelvic ring injury causing an arrest of labor.

Among the 63 women who underwent a trial of labor, 78% achieved VD, and notably, no cases of CS were performed for the arrest of labor. This is a critical finding, as it challenges the assumption that prior pelvic ring injury predisposes patients to cephalopelvic disproportion severe enough to prevent successful labor. In contrast, 28% of CS deliveries in our cohort lacked a clearly documented indication, suggesting that factors other than objective labor dysfunction may influence delivery mode.

The observed CS rate in women with a history of pelvic ring injury in our study is consistent with prior literature, which reported a pooled CS rate of approximately 54% across 152 deliveries reported in 4 retrospective studies^2-4,12^. However, these prior studies are limited by small sample sizes and incomplete obstetric data. In 3 of the 4 studies, obstetrical information was self-reported by patients, and CS indications were not reported^2-4^. In the study by Hsu et al., 20 women underwent a trial of labor, with 4 of 6 CS indicated due to the arrest of labor^12^. This is a 20% rate of CS due to the arrest of labor compared with no occurrences in the 63 patients who underwent a trial of labor in our cohort. This discrepancy may reflect differences in patient populations, obstetric management, or evolving labor guidelines, particularly following the 2014 ACOG recommendations that support longer durations before diagnosing the arrest of labor^13^.

The presence of joint-spanning implants at the time of delivery was not an independent predictor for CS. Prior survey data indicate that 59% of obstetricians would recommend CS in a young, nulliparous patient with pelvic fixation present on imaging^14^. It is unknown if obstetricians were aware of the pelvic ring injury and implants for all patients in our cohort, as it is included in the obstetric documentation for some patients but not all. The cohort also did not include any women with symphyseal-spanning implants in place at the time of delivery, so the results cannot be applied to women in that scenario. Small case series have reported successful VD in patients with pelvic fixation, and others have found no association between retained implants and CS rates^4,12^. Vallier et al. reported on successful VD in 7 patients following operative fixation of a pelvic ring injury with symphyseal plates or SI joint-spanning implants present at the time of delivery^4^. At another institution, Hsu et al. reported no difference in CS rate between patients with or without pelvis joint-spanning implants^12^. The findings of our study support a trial of labor in women with SI joint-spanning implants in place that do not have other indications for CS.

The presence of post-traumatic pelvic ring deformity was also not associated with CS in our cohort. These findings differ from Hsu et al., who reported higher CS rates for those with ≥1-cm pelvic asymmetry value (73% versus 48%), with 2 of 4 patients with the arrest of labor having an asymmetry value of >2 cm^12^. Looking specifically at this radiographic parameter in our study, CS rate with pelvic asymmetry value < 1 cm was 62% (45 of 73 deliveries) and ≥ 1 cm was 55% (26 of 47). The difference in these findings may be based on variable hospital practices and/or patient ethnicity. The definition of arrest of labor has changed over time, and shorter intervals were initially used and may still be in practice at some hospitals before and since the release of the 2014 ACOG clinical practice guidelines that include more extended timelines before assigning this diagnosis^13^. In addition, pelvis shape varies across the human population, and the effect of pelvic ring deformity will depend on preexisting structure^15^. There is likely a threshold of pelvic ring deformity that is a risk factor for arrest of labor, but we were unable to identify it in our study.

This study has several limitations. Decision-making for mode of delivery during childbirth is complex and often multifactorial. The retrospective design limits the CS indications to what was documented and does not fully represent the discussion between the patient and physician(s). The scheduled CS delivery records with no clear indications in our patient cohort frequently mentioned concerns regarding the prior pelvic ring injury, but it is unknown what recommendations were made or if the scheduled CS was based on physician or patient preference. The relatively small number of patients with prior pelvic ring injuries who underwent a trial of labor in this study limits our accuracy in predicting the true rate of labor arrest for this population overall. Last, this study only included women delivering a child at a single institution in North America and these findings may not be generalizable to other practice settings or populations.

Women with a history of pelvic ring injury demonstrated a substantially higher rate of CS delivery in future pregnancies in this cohort, often without a clearly documented indication. The arrest of labor due to post-traumatic pelvic anatomic abnormalities is likely an infrequent event, but additional studies are needed to better identify which patients may be at increased risk. Future collaborative work between obstetricians and orthopaedic surgeons is needed to determine indications for CS in patients with a history of pelvic ring injury. In the interim, orthopaedic surgeons should support women with prior pelvic ring injuries treated nonoperatively or who only have implants spanning SI joints to undergo a trial of labor in future pregnancies in a setting where cephalopelvic disproportion could be managed, although the risk is low.

## Appendix

Supporting material provided by the authors is posted with the online version of this article as a data supplement at jbjs.org (https://links.lww.com/JBJSOA/B412). This content was not copyedited or verified by JBJS.
